# Quality of life and eating habits of patients with obesity during the COVID-19 pandemic*

**DOI:** 10.1590/1518-8345.5238.3502

**Published:** 2021-11-19

**Authors:** Luciana Foppa, Ana Laura Rodriguez da Mota, Eliane Pinheiro de Morais

**Affiliations:** 1 Hospital de Clínicas de Porto Alegre, Serviço de Enfermagem Ambulatorial, Porto Alegre, RS, Brazil.; 2 Universidade Federal do Rio Grande do Sul, Escola de Enfermagem, Porto Alegre, RS, Brazil.

**Keywords:** Quality of Life, Obesity Management, Nursing Assessment, Patient Care Planning, Pandemic, Patient-Centered Care, Calidad de Vida, Manejo de la Obesidad, Evaluación en Enfermería, Planificación de Atención al Paciente, Pandemia, Atención Dirigida al Paciente, Qualidade de Vida, Manejo da Obesidade, Avaliação em Enfermagem, Planejamento de Assistência ao Paciente, Pandemia, Assistência Centrada no Paciente

## Abstract

**Objective:**

to verify the quality of life and eating habits of patients with obesity during the COVID-19 pandemic.

**Method:**

cross-sectional study with 68 outpatients, candidates for bariatric surgery, at university hospital in the Southern Brazil. Data collection was carried out by telephone, with questions about the profile of the participants and social distancing; questionnaires on quality of life and eating habits were also used. The data analysis, the logistic regression model, Spearman correlation, Mann-Whitney U and Student t-tests were used for independent samples.

**Results:**

the general quality of life was 57.03 points and the eating habit with the highest score was cognitive restraint (61.11 points). Most patients (72.1%) were socially distancing themselves and 27.9% had not changed their routine. The chance of isolation was 3.16 times greater for patients who were married. There is a positive correlation between the domains of the Quality of Life questionnaire and cognitive restraint from the questionnaire about eating habits.

**Conclusion:**

we found that the participants tended to have a better quality of life as cognitive restraint increased.

## Abstract (es)

**Objetivo:**

evaluar la calidad de vida y la conducta alimentaria de los pacientes con obesidad durante la pandemia de COVID-19.

**Método:**

estudio transversal con 68 pacientes atendidos en un servicio ambulatorio de cirugía bariátrica de un hospital universitario del sur de Brasil. La recolección de datos se realizó por vía telefónica, con preguntas sobre el perfil de los participantes y el distanciamiento social; también se utilizaron cuestionarios sobre calidad de vida y conducta alimentaria. Para el análisis de los datos se utilizó el modelo de regresión logística, la correlación de Spearman, las pruebas de la U de Mann-Whitney y la t de Student para muestras independientes.

**Resultados:**

la calidad de vida general fue de 57,03 puntos y la conducta alimentaria con mayor puntuación fue la restricción cognitiva (61,11 puntos). La mayoría de los pacientes (72,1%) mantenía el distanciamiento social y el 27,9% no habían cambiado la rutina. La probabilidad de adherir al aislamiento fue 3,16 veces mayor para los pacientes casados. Existe una correlación positiva entre los dominios del cuestionario de calidad de vida y la restricción cognitiva de las preguntas sobre la conducta asociada a los hábitos alimentarios.

**Conclusión:**

se verificó que los participantes tendían a tener una mejor calidad de vida a medida que aumentaba la restricción cognitiva.

## Abstract (pt)

**Objetivo:**

verificar a qualidade de vida e o comportamento alimentar de pacientes com obesidade durante a pandemia por COVID-19.

**Método:**

estudo transversal com 68 pacientes atendidos em ambulatório de cirurgia bariátrica em hospital universitário do sul do Brasil. A coleta de dados foi realizada por telefone, com perguntas sobre o perfil dos participantes e o distanciamento social; também foram utilizados questionários de qualidade de vida e de comportamento alimentar. Para a análise de dados, foram utilizados o modelo de regressão logística, a correlação de Spearman e os testes U de Mann-Whitney e *t* de Student, para amostras independentes.

**Resultados:**

a qualidade de vida geral foi de 57,03 pontos e o comportamento alimentar que apresentou maior pontuação foi a restrição cognitiva (61,11 pontos). Grande parte dos pacientes (72,1%) estava fazendo distanciamento social e 27,9% não haviam mudado a rotina. A chance de fazer isolamento foi 3,16 vezes maior para os pacientes que estavam casados. Existe uma correlação positiva entre os domínios do questionário de qualidade de vida e a restrição cognitiva das perguntas sobre o comportamento associado ao hábito alimentar.

**Conclusão:**

verificou-se que os participantes apresentaram tendência em ter uma melhor qualidade de vida conforme a restrição cognitiva aumentava.

## Introduction

COVID-19 is a disease caused by a new virus in the *Coronaviridae* family and had its first epicenter in the city of Wuhan, Hubei province, China, at the end of 2019^(1)^. Due to its easy transmission, in just a few weeks the main world health agencies were already looking to understand the consequences that this virus causes in the human body and what could be the best measures to control the pandemic^(1-2)^.

The Severe Acute Respiratory Syndrome - Coronavirus 2 (SARS-CoV-2), similar to other viruses in its family, such as the Middle East Respiratory Syndrome (MERS-CoV) and the Severe Acute Respiratory Syndrome (SARS-CoV), characterizes by respiratory tract infection. Its main form of transmission is from person to person or from object to person, through droplets of saliva and secretions^(3-4)^. COVID-19 demands worldwide attention due to its lethality and severity, ranging from less complex cases, with flu-like symptoms, to the most critical ones, which can progress to respiratory failure, septic shock, among other complications^(5-6)^.

As cases increased in Brazil, health authorities implemented practices to control the virus and the main measures established were social distancing (SD) and hand hygiene with alcohol gel in public spaces. Despite resistance from the federal government and the population, the strategies mentioned above are the most effective against the spread of the virus so far, by hindering the high transmissibility of the disease^(7)^.

In the state of Rio Grande do Sul (Brazil), the first official social distancing guidelines were enacted on March 16, 2020, having been recommended only to the risk group, until then composed of older adults aged over 60 years, pregnant women, and people with low immunity. In May 2020 a new decree was instituted, in the state, presenting a model of guidelines to the entire population^(8-9)^.

With the advancement of discoveries about COVID-19, patients with obesity were included in the risk group, making the SD of this group even more significant, due to the significant increase of the disease morbidity and mortality in individuals with obesity^(10)^. The role of this virus in patients with a body mass index (BMI) greater than 40 kg/m^2^ is still under analysis: it is understood that there may be greater complications due to the profile of comorbidities usually presented in this population, such as hypertension, type 2 diabetes mellitus, dyslipidemia and obstructive sleep apnea syndrome (OSAS)^(11-12)^.

Obesity is also correlated with a worsening in the quality of life of individuals, as the higher their BMI, the greater the possibility that the patient will experience pain, functional incapacities and decreased muscle strength^(13)^. Thus, the assessment of this relationship can be done through an instrument that analyzes several domains of quality of life, which has adequate psychometric characteristics and requires little time to be completed: this is the WHOQOL-Bref quality of life questionnaire, already applied in a population similar to the one in this study^(14)^.

Obesity is also related to emotional and cognitive eating behavior, which is more pronounced in patients in the preoperative period of bariatric surgery, when compared to those in the postoperative period of the same surgery^(15)^. The alterations in eating habits usually present in people with obesity are the attempt to control eating, also known as cognitive restraint; lack of eating control, or loss of self-control, which implies an exaggerated consumption of food and emotional eating, or desire to eat due to emotional and external factors^(14-15)^. From this perspective, the Three Factor Eating Questionnaire (TFEQ-21) was applied to assess cognitive restriction, disinhibition and susceptibility to hunger in adults^(16)^.

In the Bariatric Surgery Program at the Hospital de Clínicas de Porto Alegre (HCPA), patient who meets the criteria for the procedure must undergo examinations, consultations and group meetings, in which they undergo health education and clinical monitoring by multi-professional. The educational group is managed by nursing professionals and has the participation of the entire program team. The strategy of creating and conducting educational groups for patients with obesity contributes to the exchange of experiences and difficulties faced due to the disease, strengthening bonds between the participants, thus becoming an important learning space for health maintenance before and after bariatric surgery. In several studies, researchers have observed that there is a positive relationship between frequent multiprofessional monitoring and the presence of an emotional support network for the mental and physical health of these patients^(15,17)^.

It is believed that patients who participate in these groups, receiving guidance and continuous monitoring by a multidisciplinary team and developing bonds with other participants and professionals, were more committed to weight loss. Therefore, we proposed to investigate how the quality of life and eating behavior of candidates for bariatric surgery presented themselves during the restriction measures imposed by the pandemic. Thus, the aim of the study is to verify the quality of life and eating habits of patients with obesity during the COVID-19 pandemic.

## Method

### Type of study

Cross-sectional descriptive study, guided by the STROBE guideline (Strengthening the Reporting of Observational Studies in Epidemiology), which contains items that should be included in observational studies^(18)^.

### Study location

The study was carried out at the Hospital de Clínicas de Porto Alegre, Rio Grande do Sul (RS), Brazil. Every year, at this hospital, there are approximately 567,784 individual and group consultations in the offices. The bariatric surgery outpatient of the research institution is attended by the following professionals: physicians (surgeons, endocrinologists, psychiatrists, pulmonologists and cardiologists), nurses, social workers, nutritionists, psychologists and physical education professionals.

### Time course

Data collection was carried out in June 2020.

### Population

The population consisted of participants of the Bariatric Surgery Program at the Hospital de Clínicas de Porto Alegre, Rio Grande do Sul, Brazil.

### Sample

To select the study participants, intentional sampling was used, consisting of patients who were undergoing follow-up in the group entitled “*Mudança de Estilo de Vida*” (MEV - Change in Lifestyle) before the institution’s suspension of group activities, as recommended by the World Health Organization, on March 17, 2020.

Before contingency measures were taken at the hospital, six groups were maintained simultaneously, with a total of 94 patients. To participate in the study, the patient should have participated in the last meeting before the suspension of group activities by the institution. Patients who had already undergone bariatric surgery and those who did not answer the calls after three attempts, were excluded. The final sample had 68 participants.

### Study variables

To facilitate filling in the participants’ responses, an online form was created to collect data on the studied variables, such as: medical record number, telephone number, gender, age, marital status, weight, height, education, profession (occupation), comorbidities (hypertension, cardiovascular diseases, type 2 diabetes mellitus, psychiatric diseases, dyslipidemia, lung diseases, obstructive sleep apnea syndrome), in addition to the WHOQOL-Bref quality of life questionnaire, the Three Factor Eating Questionnaire (TFEQ-21) and questions about SD. Among the questions, six were closed and one, presented below, was open: “what does the pandemic represent for you?”. The form was filled out by the researchers during the calls and was prepared online, in order to preserve the social distancing.

To calculate the BMI, the weight considered was the one on the participant’s electronic medical record, taken from the last MEV group meeting in which they had participated. Weight, height, comorbidities and telephone number were consulted in the patients’ electronic medical records.

### Data collection instrument

The WHOQOL-Bref quality of life questionnaire and the Three Factor Eating Questionnaire (TFEQ-21) were translated and adapted to research in Brazil^(19-20)^. The first consists of 26 questions: questions one and two refer to the patient’s quality of life in general; while the other 24 are divided into four domains, namely, physical (questions 3, 4, 10, 15, 16,17 and 18), psychological (questions 5, 6, 7, 11, 19 and 26), social relations (questions 20, 21 and 22) and environment (questions 8, 9 12, 13, 14, 23, 24 and 25). Domain scores are converted to a scale from 0 to 100: the higher the score, the better the quality of life^(19)^.

The TFEQ-21 questionnaire, on the other hand, consists of 21 questions and determines the degrees of cognitive restraint (CR), emotional eating (EE) and uncontrolled eating (UE). It consists of nine questions about UE (3, 6, 8, 9, 12, 13, 15, 19 and 20), six about CR (1, 5, 11, 17, 18 and 21), and six about EE (2, 4, 7, 10, 14 and 16). The mean of each of the behavior variables was calculated and transformed into a scale from 0 to 100 points: high scores indicate higher UE, CR and EE^(20)^.

### Data collect

Due to the measures implemented to reduce the transmission of COVID-19, data collection took place through telephone contact with the participants. The calls were made by the researchers during business hours, that is, between 8 AM and 6 PM. Patients were asked about their interest in participating in the survey by telephone and whether they were available to answer questions on that call or whether they preferred to schedule another opportunity. The calls were recorded and the participant was asked to say the date and the following sentence: “I, (the person says his name), accepted to participate in the survey on quality of life and eating behavior during the pandemic.” The recordings were archived and will be kept for five years in a safe place, by the researchers. The application of the questionnaires lasted, on average, 19 minutes.

The project included a pilot plan, which aimed to identify possible errors in the typing of the questionnaires and reduce bias. The pilot plan was carried out with four patients who had already completed the group meetings for more than six months and were not part of the study sample.

### Data analysis

Data analysis was performed using the Statistical Package for Social Sciences (SPSS) software, version 22.0. Categorical variables were described by absolute number and percentile, and continuous variables, described by mean and standard deviation, if normally distributed—otherwise, data were described as median and interquartile range. The normality assumption was assessed using the Shapiro-Wilk test. For the results of the open question, a descriptive analysis of the data was performed through frequency distribution. Each word was sorted with a number and analyzed by its absolute frequency, highlighting the words that occurred in greater numbers. The analysis performed using the logistic regression model were: the association of social distancing measures in relation to sex and marital status, the domains of the quality of life questionnaire and the behavior associated with eating habits. All variables with p<0.30 in the univariate analysis were included in the multivariate model. To verify the association between social distancing and the domains of the questionnaire on quality of life and behavior associated with eating habits, the Mann-Whitney U and Student’s t-tests were applied for independent samples. For the correlation between the domains of the questionnaires, Spearman’s correlation was used. The level of statistical significance was 5%.

### Ethical aspects

The study was approved by the institution’s ethics committee, via *Plataforma Brasil*, under number CAAE 31651020100005327, contemplating the prerogatives announced in the Resolution 466/2012 of the National Health Council. The researchers followed the telephone call script to invite them to participate in research at the institution, which contains three options for the participant to receive and send the free and informed consent term (email, Whatsapp or text message) and, according to each one’s preference, the document was sent. When handling the information, the researchers preserved the anonymity of the participants during data processing and publication.

## Results

Before the hospital’s contingency measures, there were 94 patients undergoing follow-up in the MEV group; of these, 18 participants missed the last meeting, three had already undergone bariatric surgery and five did not answer the calls after three attempts. With that, we obtained a sample of 68 participants. As for the sociodemographic data of the sample, 52 patients (76.5%) were female, 41 (60.3%) were married or had a stable relationship, 25 (36.8%) had a paid occupation and 22 (32.4%) had incomplete primary education.

Morbid obesity was predominant in the sample, with 64 patients (94.1%) and the most prevalent comorbidities were hypertension, in 51 of them (75%), psychiatric diseases, in 30 of the patients (44.1%) and type 2 diabetes mellitus, in 27 (39.7%).

The result of general quality of life was 57.03 points and the eating behavior that presented the highest score was cognitive restraint, with 61.11 points. In Table 1, in addition to the detailed characteristics of the sample, there are the scores found for each of the domains of the quality of life questionnaire (WHOQOL-Bref), as well as the behavior associated with eating habits (TFEQ-21).

**Table 1:** Sociodemographic characteristics and scores assigned in the questionnaires on quality of life and behavior associated with eating habits of the study participants (n=68). Porto Alegre, RS, Brazil, 2020

Characteristic*	n= 68
Age (years)	45 (11)
Sex Female Male	52 (76.5) 16 (23.5)
Body Mass Index (kg/m^2^)	49.32 (7.4)
Comorbidities^†^ Hypertension Psychiatric Illnesses Type 2 Diabetes Mellitus Dyslipidemia Cardiovascular diseases Lung diseases Obstructive Sleep Apnea Syndrome	51 (75) 30 (44.1) 27 (39.7) 8 (11.7) 7 (10.2) 6 (8.8) 2 (2.9)
Marital Status Married or in a stable relationship Single or without a steady partner	41 (60.3) 27 (39.7)
Profession (occupation) Housewife Student Paid occupation No paid occupation/unemployed On sick pay Retired on disability	15 (22.1) 1 (1.5) 25 (36.8) 7 (10.3) 12 (17.6) 8 (11.8)
Domains of quality of life General quality of life Environmental Social Psychological Physical	55.43 (12.63) 55.23 (15.78) 62.99 (20.33) 51.34 (15.77) 52.15 (11.69)
Domains of the Three Factor Eating Questionnaire Uncontrolled eating Emotional eating Cognitive restraint	30.66 (22.5) 36.84 (30.51) 60.70 (17.86)

* Continuous variables described by mean and standard deviation and categorical by absolute number and percentile;

† More than one response to this variable was computed

When asked about COVID-19, 63 participants (92.6%) had not yet become infected, 4 (5.9%) had suspected contagion, but underwent the test and obtained a negative result and only 1 (1. 5%) was suspected of contagion, waiting for the test result, at the time of data collection. In the question about contact with someone who has had COVID-19 confirmed, 63 (92.6%) had not had contact with people infected with the virus and 5 (7.4%) reported having had contact with a family member, friend, or neighbor with positive for coronavirus.

When asked about social distancing, 49 (72.1%) participants had adhered to the measures at the time of data collection and 19 (27.9%) had not changed their routine and continued to move about as before. When analyzing the association between adhering or not to SD and marital status, a significant difference (p=0.02) was found. Of the married people or those with a stable relationship, 82.9% were following the measures, while among the single individuals or those without a steady partner, 55.6% were following it. Regarding sex, there was a significant difference (p=0.01). Of the females, 80.8% were adhering to social distancing; while among the males, 43.8% were adhering to it, at the time of the survey.

In the comparison analysis of the quality of life questionnaire and the behavior by SD, there was no statistically summarized difference. Table 2 shows the association of this measure with the quality of life questionnaire and behavior associated with eating habits, as well as an association of social distancing with sex and marital status of the 68 participants.

**Table 2:** Association of social distancing with sex, marital status, the domains of the quality of life questionnaires and behavior associated with eating habits of the research participants (n=68). Porto Alegre, RS, Brazil, 2020

Characteristic*	With social distance	No social distance	p-value
Sex Female Male	42 (80.8) 7 (43.8)	10 (19.2) 9 (56.3)	0.01^9†^
Marital Status Single or without a steady partner Married or in a stable relationship	15 (55.6) 34 (89.2)	12 (44.4) 7 (17.1)	0.02^7†^
Domains of quality of life General quality of life Environmental Social Psychological Physical	55 (12) 54 (15) 63 (20) 50 (15) 51 (11)	56 (14) 56 (16) 60 (20) 53 (17) 54 (12)	0.788^‡^ 0.657^‡^ 0.619^‡^ 0.574^‡^ 0.381^‡^
Domains of the Three Factor Eating Questionnaire Uncontrolled eating Emotional eating Cognitive restraint	29 (20) 34 (29) 60 (17)	33 (27) 43 (33) 60 (18)	0.573^‡^ 0.322^§^ ^0^.891‡

* Continuous variables described by mean and standard deviation, and categorical, by absolute number and percentile;

† Yates continuity correction test;

‡ Student’s t test for independent samples;

§ Mann-Whitney U Test

Table 3 shows the SD univariate and multivariate logistic regression model for the variables sex and marital status, domains of the quality of life questionnaire and behavior associated with eating habits. It can be said that for those who were married or in a stable relationship, the chance of socially distancing themselves was 3.16 times greater than for those who were single or without a steady partner.

**Table 3:** Logistic regression of social distancing with sex, marital status, domains of the quality of life questionnaire and behavior associated with eating habits (n=68). Porto Alegre, RS, Brazil, 2020

Characteristic	OR (CI)*	*p-value* ^†^	Adjusted OR (CI)^‡^	*p-value* ^†^
Sex Male	0.521 (0.164-1.655)	0.269	0.585 (0.175-1.147)	0.382
Marital Status Married	3.302 (1.148-9.499)	0.027	3.166 (1.091-9.186)	0.034
Domains of QL^§^ General QL^§^ Environmental Social Psychological Physical	0.994 (0.954-1.035) 0.97 (0.965-1.030) 1.006 (0.981-1.032) 0.983 (0.950-1.016) 0.988 (0.945-1.032)	0.757 0.872 0.646 0.318 0.592		
Domains TFEQ-21^||^ Uncontrolled eating Emotional eating Cognitive restraint	1.000 (0.978-1.023) 1.003 (0.986-1.020) 0.997 (0.969-1.026)	0.995 0.743 0.832		

* OR = Odds Ratio; CI = confidence interval;

† Significance of the test;

‡ OR adjusted for the other predictors of the model (only variables with p < 0.30 in the univariate analysis were included in the multivariate model);

§ QL = Quality of life;

|| TFEQ-21 = Three Factor Eating Questionnaire

Table 4 shows the correlation analysis between the domains of the quality of life questionnaire and behavior associated with eating habits. There is a weak to moderate negative correlation between the domains of the quality of life questionnaire and the UE and EE domains of the questions about behavior associated with eating habits; showing that as the quality of life decreases, the UE and EE tend to increase.

**Table 4:** Correlation between domains of the quality of life questionnaires and behavior associated with eating habits (n=68). Porto Alegre, RS, Brazil, 2020

Domain	Uncontrolled eating		Emotional eating		Cognitive restraint	
	r*	*p-value* ^†^	R	*p-value* ^†^	r	*p-value* ^†^
General quality of life	-0.45	<0.001	-0.38	0.001	0.21	0.077
Environmental	-0.28	0.019	-0.25	0.037	0.20	0.103
Social	-0.40	0.001	-0.36	0.002	0.19	0.880
Psychological	-0.49	<0.001	-0.35	0.003	0.27	0.025
Physical	-0.31	0.008	-0.33	0.006	0.34	0.004

* r = Correlation coefficient;

† Spearman correlation

The open question, about what the pandemic represented to the participant, 55.6% answered that it represented the fear of contamination and 44.6% reported being afraid of contaminating their families. Other feelings mentioned by the participants were terror, chaos, and sadness.

## Discussion

In this study, we found that the association between SD, the quality of life and eating behavior of obese individuals, during the pandemic, did not present a statistically significant difference. However, it was observed that as quality of life decreases, emotional eating (EE) and uncontrolled eating (UE) tended to increase. The data also showed a significant relationship between adherence to social distancing and marital status; the chance of social distance was greater for those who were married or in a stable relationship.

Most participants were female and were in a stable relationship or married, characteristics that are consistent with recent studies, in which researchers assessed the profile of patients awaiting bariatric surgery^(21-22)^. Although obesity affects more males, women are the ones who most seek the surgical procedure as a treatment^(23)^, as there is greater social pressure for a healthy body or within the standards of beauty; additionally, among women, there is better understanding and adherence to health care, which can boost the search for bariatric surgery^(21,24)^. It is assumed that women have a better understanding of the health risks caused by obesity, are more concerned about their well-being and are more adept at health guidelines, supporting their better adherence to social distancing.

In our study, the most prevalent associated diseases were hypertension, type 2 diabetes mellitus and psychiatric diseases, in agreement with the literature^(21,23,25)^. This comorbidity profile, commonly seen in obese patients, confers a greater risk of complications when there is a diagnosis of COVID-19. It is estimated that the state of chronic inflammation and the dysregulated immune response increase the chances of these patients presenting severe forms of the disease and dying^(26)^.

In terms of income, almost a quarter of the participants are in paid employment. However, the variables “Housewife” and “On sick pay” were also prevalent. Discrimination against people with obesity causes absenteeism and dismissal from work, reinforcing social isolation behaviors and aggravating the mental health of workers with obesity^(27)^. With the pandemic, the distance work modality became necessary to prevent the transmission of the virus, however, it created other factors that can compromise the quality of life of these patients: feeling of isolation and sedentarism^(28)^.

Within the participants, the quality of life observed was impaired. Previous studies have reported the association between obesity and quality of life, showing negative aspects; since the greater the severity of the disease and its comorbidities, the greater the damage to the quality of life of patients^(14,29)^. There are still few studies that address the consequences that the COVID-19 pandemic had on patients with obesity. Studies have shown, however, that intense psychological distress and higher levels of stress can increase risky eating behaviors and negatively affect quality of life^(30-31)^.

In the psychological domain of the quality of life questionnaire, participants had lower scores; enabling us to assume that being overweight imposes obstacles to a healthy life, which go beyond a debilitated physical condition. Stigma acts as an important factor, presenting itself in the form of verbal and physical violence against these individuals^(32)^. People with obesity, especially women, perceive this discrimination and, as a consequence, present feelings of inferiority, embarrassment, sadness, frustration and low self-esteem, which have everlasting repercussions^(28)^. In the pandemic context, complications of psychiatric illnesses are described, in which the levels of stress, anxiety, and depression were aggravated in individuals with chronic diseases^(33)^. There is a high incidence of psychiatric illnesses in people with obesity^(23,25)^; almost half of the study participants presented some form of it.

Regarding the responses obtained in the social, environmental, and physical domains of the quality of life questionnaire, we found that they corroborate data from studies in which researchers evaluated the quality of life of patients in the pre- and post-bariatric surgery periods. Patients showed dissatisfaction with the physical environment, related to safety and leisure issues^(14,29,34)^. It is suggested that the quality of life of candidates for bariatric surgery is impaired, as excess weight negatively affects self-esteem^(14)^.

Regarding the eating behavior of participants during the pandemic, it was found that cognitive restraint (CR) was the domain with the highest score and that this tends to positively influence quality of life. CR has been shown to be the most prevalent behavior among obese individuals^(35)^, however, there are contradictions in how it is associated with weight gain. CR is expressed in the understanding that it is necessary to eat less than desired and in a healthier way. However, in obese individuals, it is more difficult to put this into practice when compared to overweight or eutrophic individuals^(36-37)^.

The association between CR and other behaviors was not evaluated in this study, but other studies shows that CR may be related to UE, in which the individual needs to eat even after feeling full^(38)^. In addition, CR can also be associated with UE, as a form of defense against an unpleasant emotion, for example, sadness^(15)^. In this relationship, the patient understands that he needs to control or lose weight, but when he finds himself with a negative stimulus or goes through situations of great stress, he ends up eating in an exaggerated and compensatory way, generating a feeling of guilt and frustration^(36)^.

Participants had higher scores in UE behavior when compared to EE behavior and both tend to negatively influence their quality of life. In recent studies, it was observed that the population, during social distancing measurements, did not show significant changes in CR, but there was a significant increase in UE and EE, resulting in their weight gain, especially in women^(38-39)^. In obese people, the risk of worsening symptoms of these behaviors is even greater. However, an assessment of the impact of social isolation on obesity showed that participants did not obtain a significant increase in BMI or worsening of symptoms^(40)^.

By the end of data collection, no participant had tested positive for COVID-19, with few suspected cases to be confirmed. Most were complying with SD and following hygiene measures. In some of the participants’ reports, intense fear of contamination and fear of contaminating their families were observed. Fear, insecurity and negative feelings have been commonly observed in people who adhere to some form of SD during the pandemic^(41)^. Some studies agree that the government plays an important role in the population’s emotional control in times of pandemic; through the development of public actions, the population’s stress level can be reduced and social distancing can be ensured. As an example, we can mention the provision of information from official sources to the population, the availability of social support and good communication with the health sectors^(42-43)^.

The data collected at the time of the pandemic corroborate studies that assess the response of people with obesity to situations of great stress. Future studies should be carried out to understand how the relationship between CR and other eating behaviors of people with obesity will be in the long term after the pandemic and also how social distancing will affect the quality of life of individuals who already lack a healthy lifestyle.

Bariatric surgery candidates expect to live a healthier life after the surgical procedure. For this to be possible, the interconnection of environmental, physical, mental and social aspects that vary according to each individual must be worked out with the patient. Individuals awaiting bariatric surgery have specific needs, as well as particular clinical and behavioral characteristics, which affect the way they relate to the environment.

The present study has some limitations that interfere with the generalization of the results. The population belongs to a certain group, with distinct social and economic characteristics, when compared to people with normal BMI. Patients were selected from the preoperative bariatric surgery group, in which they receive guidance on the benefits of changing their lifestyle and the information is self-reported. Another weakness that can be considered in the study is that, during data collection, the state of Rio Grande do Sul had not yet reached the peak of contamination and the population still adjusted to the new norms and routine^(44)^. This fact needs to be considered to understand the seriousness of the impact that the pandemic will have on obese people, in the long term. Additionally, it would be interesting to evaluate in detail the daily activities and meals of the participants in order to determine how much this affects the variables studied, especially eating behavior.

Nursing, as a part of the multidisciplinary team of programs for bariatric surgery, plays an important role in the humanized care of patients before and after surgery. During the pandemic, this action proved to have a great impact on maintaining the health of these patients. With this study, a basis is created for nurses to contribute positively to the current problem developed by the pandemic, focusing on educational actions so that patients continue to adhere to the treatment and on facing the changes that the pandemic brought to their lifestyle. Furthermore, the role of nursing in this area is still not widespread, as well as national publications on the subject. Therefore, this study contributes to the science of nursing and emphasizes the need to value the professional nurse in the role of managing the care of patients with obesity.

## Conclusion

In our study, it was found that during the COVID-19 pandemic, participants tended to have a better quality of life as their cognitive restraint (CR) increased. However, the behavior of EE and UE can negatively influence the well-being of individuals with obesity, demonstrating that psychological and physical factors can impact their quality of life.

The investigation of quality of life and eating behavior during the COVID-19 pandemic can serve as a starting point for the adoption of educational and cognitive-behavioral approach strategies for patients with obesity. However, further studies on the subject would be needed to assess the real impact of the COVID-19 pandemic on the quality of life and eating behavior of these patients.
